# Prebiotic Effects of *Lycium barbarum* Polysaccharides on Gut Microbiota and Short‐Chain Fatty Acids Production in Wilson's Disease: An In Vitro Fermentation Study

**DOI:** 10.1002/fsn3.71815

**Published:** 2026-04-21

**Authors:** Shuzhen Fang, Kangyi Zhang, Xiang Fang, Danqing Liu, Yulong Yang, Guijie Chen, Wenming Yang

**Affiliations:** ^1^ First Affiliated Hospital of Anhui University of Traditional Chinese Medicine Hefei China; ^2^ Center for Xin'an Medicine and Modernization of Traditional Chinese Medicine Institute of Health and Medicine Hefei Comprehensive National Science Center Anhui China; ^3^ Key Laboratory of Xin'an Medicine Ministry of Education Hefei China; ^4^ National Key Laboratory for Tea Plant Germplasm Innovation and Resource Utilization Anhui Agricultural University Hefei China

**Keywords:** gut microbiota, in vitro fermentation, *Lycium barbarum* polysaccharides, short‐chain fatty acids, wilsons disease

## Abstract

Wilson's disease (WD) is a rare autosomal recessive disorder characterized by impaired copper metabolism and progressive multi‐organ damage. Emerging evidence suggests that gut microbiota dysbiosis and reduced microbial fermentation capacity may contribute to the progression of liver diseases. However, whether the gut microbiota can serve as a therapeutic target in WD remains unclear. This study aimed to investigate the modulatory effects of *Lycium barbarum* polysaccharides (LBPs), a bioactive component of the traditional Chinese medicine Gan‐Dou‐Fu‐Mu decoction, on gut microbiota composition and short‐chain fatty acids (SCFAs) production in WD, and to explore their potential as microbiota‐targeted prebiotic interventions. WD‐associated microbiota exhibited decreased microbial diversity and significantly lower SCFAs production, particularly acetic acid and total SCFAs, compared to healthy controls. LBPs supplementation significantly increased the relative abundances of beneficial genera, including *Lactobacillus*, *Lacticaseibacillus*, and *Paraeggerthella*, as well as SCFAs‐producing species such as *Bacteroides fragilis* and *Lactobacillus delbrueckii* . Notably, LBPs markedly enhanced acetic acid and total SCFA concentrations in WD microbiota, outperforming the standard treatment agent penicillamine. These findings highlight the promising prebiotic potential of LBPs and support their application as a novel microbiota‐targeted adjunctive therapy for WD.

## Graphical abstract

Schematic overview of the experimental workflow investigating the effects of LBPs on gut microbiota.

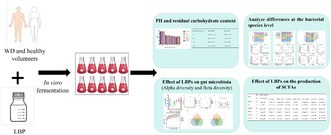

## 1 Introduction

Wilson's disease (WD) is a rare autosomal recessive disorder of copper metabolism, first systematically described by Kinnier Wilson in 1912. The disease is caused by mutations in the ATP7B gene located on chromosome 13q14.3, which impair copper transport and excretion via bile. Consequently, excess copper accumulates in vital organs such as the liver, brain, kidneys, and cornea, leading to progressive hepatic, neurological, and psychiatric symptoms (Elmehrath et al. 2024; Ryan et al. 2019; Shribman et al. 2021). As one of the few treatable neurogenetic conditions, early diagnosis and intervention are crucial to prevent irreversible organ damage and improve patient outcomes. Current treatment regimens primarily rely on copper‐chelating agents, such as penicillamine and dimercaptopropanesulfonate, which can effectively reduce copper burden. However, clinical limitations remain significant: up to 20% of patients experience neurological deterioration after chelation initiation, and adverse effects such as hypersensitivity, immunological complications, or overtreatment‐induced copper deficiency are not uncommon (Bandmann et al. 2015). These challenges highlight the need for adjunctive or alternative therapeutic strategies with improved safety and broader biological benefits.

Recent research has increasingly demonstrated that gut microbiota dysbiosis is closely linked to a wide range of human diseases, including metabolic disorders, neurodegenerative conditions, autoimmune diseases, and liver dysfunction (Chen et al. 2023; Chen et al. 2025). The gut microbiota exerts systemic effects not only by shaping local intestinal immunity and metabolism, but also through the production of microbial metabolites, among which short‐chain fatty acids (SCFAs) play a particularly critical role (Sanz et al. 2025). Given the central role of SCFAs in mediating host–microbiota interactions and maintaining physiological homeostasis, strategies aimed at enhancing SCFA production have attracted considerable attention (Mann et al. 2024). Among these, dietary polysaccharides have emerged as potent prebiotic agents capable of selectively modulating gut microbial composition and metabolic activity (Sanz et al. 2025). The prebiotic potential of polysaccharides has been well‐documented in various metabolic and inflammatory diseases (Mann et al. 2024); however, their role in the context of Wilson's disease remains largely unexplored. Whether polysaccharides can beneficially reshape the gut microbiota of WD patients and restore SCFAs‐generating capacity is an important question with both mechanistic and therapeutic implications.

Our research group has previously demonstrated the clinical efficacy and safety of the traditional Chinese herbal formula Gan‐Dou‐Fu‐Mu Decoction (GDFMD) in treating WD (Tang et al. 2023). Within this formulation, *Lycium barbarum* (goji berry) serves as the monarch herb. Its key bioactive component, *Lycium barbarum* polysaccharides (LBPs), has attracted growing interest due to its antioxidant, anti‐inflammatory, immunomodulatory, and neuroprotective properties (Wang et al. 2019; Xiao et al. 2022; Zhang et al. 2020). Importantly, LBPs are non‐digestible polysaccharides that resist enzymatic breakdown in the upper gastrointestinal tract, allowing them to reach the colon intact. There, they are fermented by gut microbiota into metabolites, including SCFAs, that can exert systemic physiological effects. As such, LBPs are considered to possess prebiotic potential, capable of selectively stimulating beneficial bacterial taxa and reshaping microbial ecology (Guo et al. 2022). Despite extensive research on the pharmacological activities of LBPs, their interactions with gut microbiota in the context of WD remain largely unexplored. Understanding these interactions is essential for elucidating the mechanistic basis of GDFMD's therapeutic effects, particularly given the emerging role of microbiota in hepatic and neurological diseases.

In this study, we aimed to investigate the prebiotic effects of LBPs on the gut microbiota of WD patients using an in vitro simulated digestion and anaerobic fermentation model, combined with high‐throughput 16S rRNA gene sequencing and SCFA quantification. This integrative approach allowed us to assess their capacity to modulate gut microbial composition and SCFA production. By comparing the responses of microbiota from WD patients and healthy individuals, and using penicillamine as a pharmacological control, we sought to clarify the microbiota‐mediated pathways through which LBPs may contribute to WD management. Ultimately, this study provides novel insights into the gut microbiota–polysaccharide interaction in WD and supports the potential of LBPs as a microbiota‐targeted adjunctive therapy.

## 2 Materials and Methods

### 2.1 Materials

LBPs were obtained from Solarbio (Beijing, China). Standards of SCFAs, including acetic acid, propionic acid, n‐butyric acid, i‐butyric acid, n‐valeric acid, and i‐valeric acid, were purchased from Aladdin Chemical Reagent Co. Ltd. (Shanghai, China). 2‐Ethylbutyric acid was obtained from Sigma Chemical Co. (St. Louis, MO, USA). Bile salts were purchased from Shanghai Ryon Biological Technology Co. Ltd. (Shanghai, China). Penicillamine was supplied by the First Affiliated Hospital of Anhui University of Traditional Chinese Medicine (Hefei, China). All other chemical reagents used in this study were of analytical grade and were purchased from Sinopharm Chemical Reagent Co. (Shanghai, China).

### 2.2 Fermentation of Gut Microbiota In Vitro

An in vitro anaerobic fermentation model was used to simulate intestinal microbial digestion and investigate the degradation of LBPs by gut microbiota according to the previous work (Chen et al. 2018). This study was approved by the Ethics Committee of the First Affiliated Hospital of Anhui University of Traditional Chinese Medicine (Approval No. 2024AH‐60‐01). Briefly, a basal nutrient medium was prepared by dissolving the following components in 1.0 L of distilled water: 2.0 g of peptone, 1.0 mg of bladed asphaltene, 2.0 mL of Tween 80, 0.02 g of hemoglobin, 10 μL of vitamin K1, 0.5 g of bile salts, 0.5 g of cysteine hydrochloride, 2.0 g of yeast extract, 0.01 g of MgSO_4_, 0.1 g of NaCl, 2.0 g of NaHCO_3_, 0.01 g of CaCl_2_, and 0.04 g of K_2_HPO_4_. The pH of the solution was adjusted to 7.0 using 0.1 M HCl, followed by sterilization via autoclaving. Fecal samples were collected from eight volunteers, including four healthy individuals (2 females and 2 males, aged 20–40 years) and four patients with WB (2 females and 2 males, aged 20–40 years). None of the participants had gastrointestinal disorders or had taken antibiotics within the past 3 months. Fresh fecal samples (5 g per individual) were mixed with 45 mL of autoclaved modified saline solution (containing 0.5 g/L cysteine hydrochloride and 9.0 g/L NaCl). The mixture was centrifuged at low speed to remove large particles, and the resulting 10% fecal suspensions from each group were pooled.

For fermentation, 1 mL of pooled fecal suspension was inoculated into 9 mL of the basal medium containing 100 mg of LBPs in sterilized conical flasks. The flasks were then immediately transferred to an anaerobic chamber and incubated at 37°C under anaerobic conditions using gas‐generating packs. Penicillamine was used as a positive control drug. The experiment included six groups based on the source of gut microbiota (healthy individuals or WB patients) and the type of treatment applied. Among them, the Control group (derived from healthy individuals) and the Model group (derived from WB patients) were fermented in vitro using only the basal nutrient medium, without the addition of any active substances. The Penicillamine_Control (PCA‐C) group and the Penicillamine_Model (PCA‐M) group were supplemented with penicillamine, using microbiota from healthy individuals and WB patients, respectively. Similarly, the LBP_Control (LBP‐C) group and the LBP_Model (LBP‐M) group received LBPs treatment and were inoculated with microbiota from healthy individuals and WB patients, respectively. Samples were collected at 6, 12, and 24 h during fermentation for subsequent analysis.

### 2.3 Determination of SCFAs

The concentrations of SCFAs, including acetic acid, propionic acid, n‐butyric acid, isobutyric acid, n‐valeric acid, and isovaleric acid, were determined at different fermentation time points using gas chromatography (GC), following the previous method (Tian et al. 2016). 2‐Ethylbutyric acid was used as the internal standard. A 2‐ethylbutyric acid solution (0.3 mg/mL) was prepared in 0.2 mol/L hydrochloric acid. For each sample, 400 μL of the fermentation supernatant was mixed with 10 μL of the internal standard solution. The mixture was filtered through a 0.45 μm membrane, and 1 μL of the filtrate was injected for GC analysis. GC analysis was performed using an HP‐INNOWAX capillary column (30 m × 0.25 mm × 0.25 μm, Agilent) equipped with a flame ionization detector (FID). Nitrogen was used as the carrier gas at a flow rate of 19.0 mL/min. The flow rates for air, hydrogen, and nitrogen makeup gas were 260, 30, and 30 mL/min, respectively. The column temperature was initially set at 100°C and held for 1 min, then increased to 180°C at a rate of 5°C/min and maintained at 180°C for 4 min. Lactic acid content was determined by high‐performance liquid chromatography (HPLC), according to the method described by Zhou et al. (2016).

### 2.4 Gut Microbiology Analysis

Genomic DNA was extracted from fecal samples using the E.Z.N.A. Stool DNA Kit (D4015; Omega Bio‐Tek, USA). 16S rRNA gene sequencing was performed by Genesky Biotechnologies Inc. (Shanghai, China). Specifically, the V3–V4 region of the 16S rRNA gene was amplified using the primers 341F (CCTACGGGNGGCWGCAG) and 805R (GACTACHVGGGTATCTAATCC) via PCR with 32 amplification cycles. PCR products were verified by 2% agarose gel electrophoresis, purified using AMPure XP beads (Beckman Coulter Genomics, Danvers, MA, USA), and quantified using a Qubit fluorometer (Invitrogen, USA). The purified amplicons were then subjected to paired‐end sequencing (2 × 250 bp) on the Illumina NovaSeq PE250 platform, following the manufacturer's protocol. Sequencing reads were assigned to individual samples based on unique barcodes. After removing the barcode and primer sequences, paired‐end reads were merged using FLASH (v1.2.8). Quality filtering of raw reads was conducted using fqtrim (v0.94) to obtain high‐quality clean tags. Chimeric sequences were removed using Vsearch (v2.3.4), and the remaining reads were denoised and dereplicated using DADA2 to generate the feature table and representative sequences.

Alpha and beta diversity analyses were performed using QIIME 2. Taxonomic classification was conducted by sequence alignment using BLAST against the SILVA database (Release 132: https://www.arb‐silva.de/documentation/release‐132/) and the NT‐16S database. Principal Component Analysis (PCA) and Principal Coordinates Analysis (PCoA) were used to assess alpha diversity and were visualized using OmicStudio tools (https://www.omicstudio.cn/tool). To identify taxa with significantly different relative abundances among groups, Linear Discriminant Analysis Effect Size (LEfSe) was applied using the Kruskal–Wallis test, Wilcoxon rank‐sum test, and linear discriminant analysis (LDA) score as parameters.

### 2.5 Statistical Analysis

Data are presented as mean ± standard error of the mean (SEM). Statistical significance of differences in the relative abundance of operational taxonomic units (OTUs) among groups was assessed using one‐way analysis of variance (ANOVA) followed by Tukey's Honestly Significant Difference (HSD) post hoc test in GraphPad Prism 8. Differentially abundant OTUs between groups were further identified using Linear Discriminant Analysis Effect Size (LEfSe). A *p*‐value of < 0.05 was considered statistically significant.

## 3 Results

### 3.1 Changes in pH and Residual Carbohydrate Content During Anaerobic Fermentation

**FIGURE 1:**
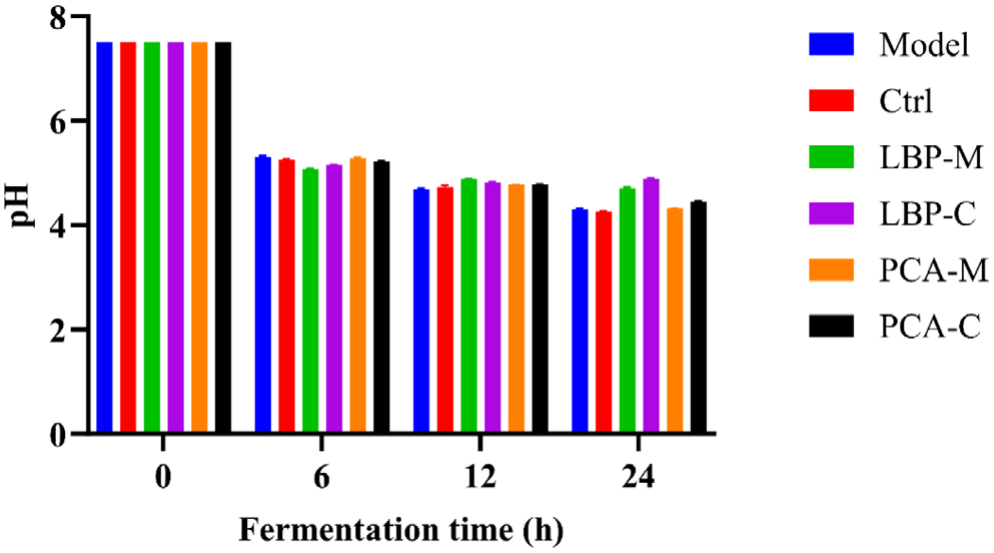
pH values of each group during anaerobic fermentation. No statistically significant differences were observed among the groups (*p* > 0.05).

The pH changes during anaerobic fermentation were monitored at 0, 6, 12, and 24 h, as shown in Figure 1. No statistically significant differences in pH were observed between the groups at the same time points. However, in all groups, the pH values decreased significantly over time compared to the initial value at 0 h (*p* < 0.05), indicating the accumulation of acidic metabolites and an overall increase in microbial fermentation activity. In parallel, the residual carbohydrate content in the fermentation broth was measured to evaluate the extent of LBPs utilization by the gut microbiota over time. As shown in Table 1, the proportion of residual carbohydrates (%) declined progressively with increasing fermentation time in both groups, suggesting that LBPs are gradually metabolized by the gut microbiota.

**TABLE 1:** Proportion of residual carbohydrates (%) at different time points during fermentation.

Fermentation time (h)	LBP‐M (%)	LBP‐C (%)
0	100.67 ± 5.51^a^	100.33 ± 3.79^a^
6 h	66.04 ± 4.33^b^	85.21 ± 4.15^b^
12 h	41.84 ± 4.92^c^	73.32 ± 1.46^c^
24 h	26.58 ± 6.29^d^	55.60 ± 0.25^d^

*Note:* Values are expressed as mean ± SD (*n* = 3). Different superscript letters within the same column indicate significant differences among time points (*p* < 0.05), determined by one‐way ANOVA followed by Tukey's post hoc test.

Interestingly, a distinct difference in utilization efficiency was observed between the microbiota from healthy individuals (LBP‐C group) and those from patients (LBP‐M group). After 24 h of fermentation, the LBP‐C group retained 55.60% ± 0.25% of the initial carbohydrate content, whereas the LBP‐M group retained only 26.58% ± 6.29%. This substantial reduction indicates that the gut microbiota from patients exhibited a more efficient ability to degrade and utilize LBPs. These findings suggest a potentially altered microbial composition or metabolic capacity in the patient group, which may enhance polysaccharide fermentation under anaerobic conditions.

### 3.2 Effect of LBPs on Gut Microbiota From WD Patients

#### 3.2.1 Alpha Diversity Analysis of Gut Microbiota

**FIGURE 2:**
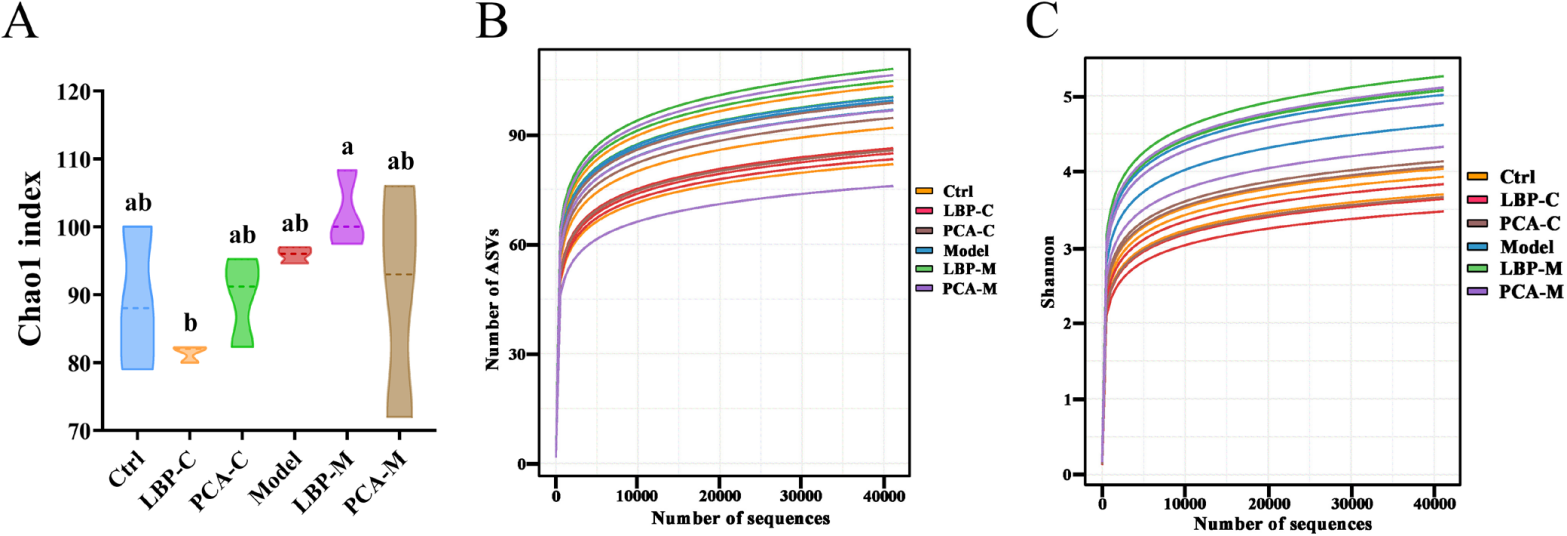
Alpha diversity of gut microbiota across different groups. (A) Chao1 index reflecting species richness. (B) Rarefaction curves and (C) Shannon index assessing sequencing depth and richness. Different letters indicate significant differences among groups (*p* < 0.05), as determined by one‐way ANOVA followed by Tukey's post hoc test.

As shown in Figure 2, each line represents an individual sample, illustrating the within‐sample (alpha) diversity of the gut microbiota in different groups. The rarefaction curves and Shannon index values plateaued across all samples, indicating that the sequencing depth was sufficient to capture the majority of microbial diversity, and that most microbial taxa present in the samples were adequately covered. No significant differences in the Chao1 index, which reflects species richness, were observed between the control and model groups, suggesting that WD itself did not cause a substantial loss in microbial richness. Similarly, the addition of LBPs or PCA alone did not significantly alter the Chao1 index when compared to the model group. However, a notable difference was observed between the LBP‐M and LBP‐C groups: the Chao1 index in the LBP‐M group was significantly higher than in the LBP‐C group (*p* < 0.05). This suggests that the gut microbiota from WD patients exhibited greater microbial richness in response to LBPs treatment compared to that from healthy individuals. This observation may be closely related to the enhanced carbohydrate utilization capacity observed in the LBP‐M group (as discussed in Section 3.1). The more efficient fermentation of LBPs by the WD‐associated microbiota could create a more favorable environment for the proliferation of a broader range of microbial species, thereby increasing richness. It is possible that certain microbial populations in the WD microbiota are more adept at metabolizing polysaccharides, leading to the enrichment of specific taxonomic groups not present or less abundant in the healthy gut microbiota.

#### 3.2.2 Beta Diversity Analysis of Gut Microbiota

**FIGURE 3:**
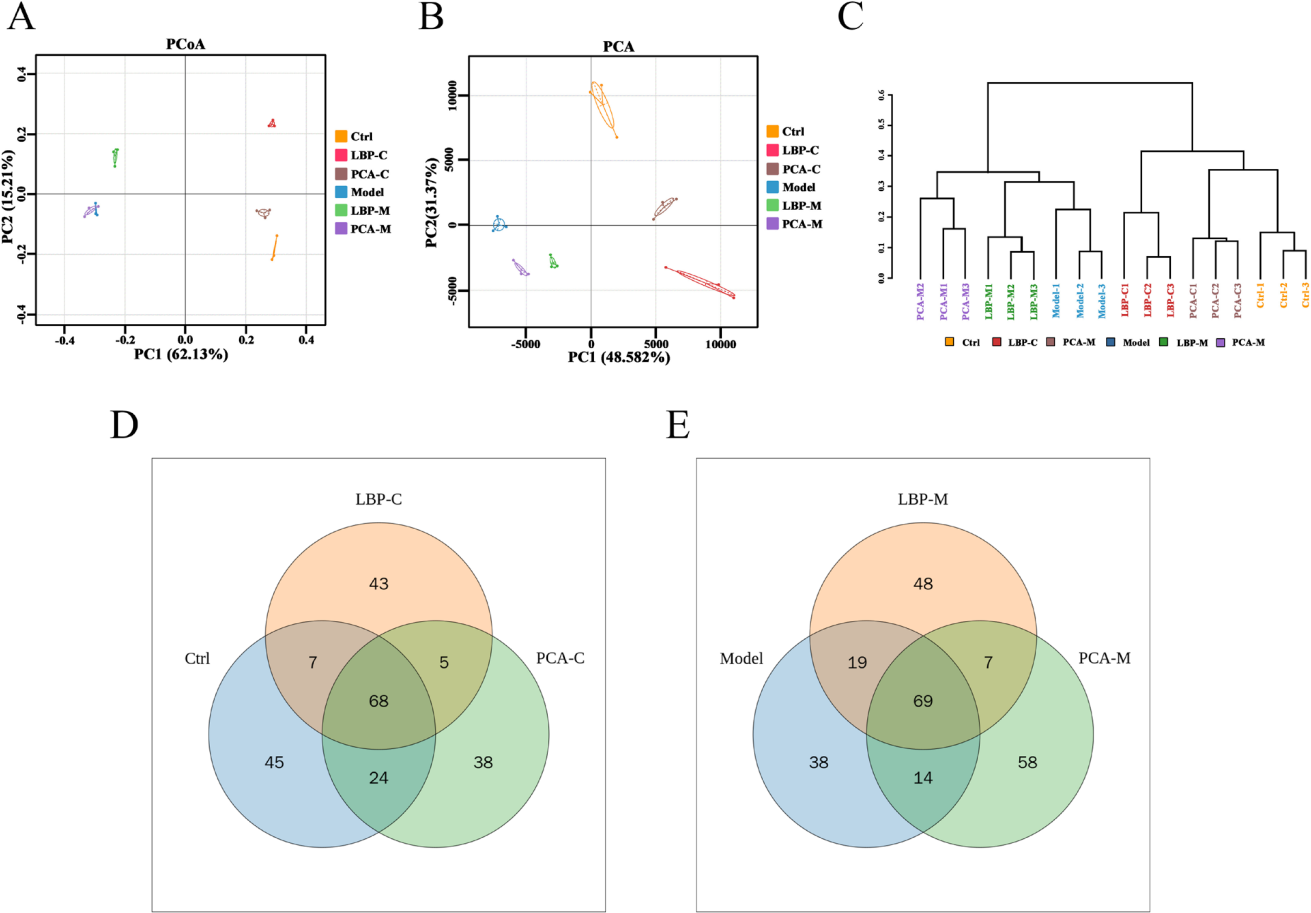
Structural differences in gut microbiota among experimental groups. (A) Principal coordinates analysis (PCoA) based on OTU‐level β‐diversity. (B) Principal component analysis (PCA) illustrating clustering patterns of microbial communities. (C) Clustering analysis based on the weighted unifrac method. (D, E) Venn diagram showing shared and unique OTUs among the Ctrl, LBP‐C, PCA‐C, LBP‐M, and PCA‐M groups.

To evaluate the overall structural differences in gut microbial communities, beta diversity analysis was performed at the OTU level. As illustrated in Figure 3, both Principal Coordinates Analysis (PCoA) and Principal Component Analysis (PCA) revealed clear separations among the experimental groups, indicating distinct microbial community structures under different fermentation conditions. PCoA plots (Figure 3A) demonstrated a pronounced separation between the Model and Control groups following anaerobic fermentation, suggesting that WD significantly alters gut microbial composition, leading to a dysbiotic state. Notably, when LBPs were used as a carbon source, a marked shift in microbial community structure was observed in both healthy individuals and WD patients. The LBP‐treated groups exhibited microbial profiles that diverged from their respective disease baselines and moved closer to the healthy control group, highlighting the strong modulatory effect of LBPs on gut microbiota composition. In contrast, penicillamine, a conventional therapeutic agent for WD, exerted only modest effects on gut microbial composition. Specifically, the PCA‐M group clustered closely with the untreated Model group, indicating that penicillamine failed to significantly reverse the dysbiotic microbial community associated with WD. This limited modulatory effect was particularly evident in WD patients, suggesting that penicillamine may not adequately address microbiota‐related components of the disease pathology.

Similar patterns were observed in the PCA analysis (Figure 3B), which further confirmed that the microbial profiles of both the LBP‐C and LBP‐M groups were more closely aligned with the Control group than those of the penicillamine‐treated groups. These findings underscore the superior capacity of LBPs to reshape gut microbial communities, suggesting a more robust and balanced prebiotic effect that operates regardless of the host's baseline microbiota status. To further characterize the microbial community structure, hierarchical clustering analysis and Venn diagram comparisons were conducted (Figure 3C). OTUs from all samples were clustered and taxonomically annotated to identify shared and unique microbial taxa. A total of 26 OTUs were found to be common across all groups, reflecting a core microbiota. However, 34 OTUs were unique to the Model group, indicative of disease‐specific microbial alterations and reduced ecological stability. In contrast, the LBP‐M group harbored 44 unique OTUs, suggesting that LBPs fermentation not only enhances microbial richness but also promotes the emergence of novel or functionally distinct taxa, possibly beneficial bacteria involved in polysaccharide metabolism, immune modulation, or gut barrier function. Taken together, these results demonstrate that WD induces profound disruptions in gut microbial diversity and composition. While penicillamine exhibits limited capacity to restore microbial homeostasis, LBPs exhibit a strong microbiota‐modulating effect, capable of partially reversing dysbiosis and promoting a more eubiotic (health‐associated) microbial environment. These findings highlight the promising potential of LBPs as a microbiota‐targeted therapeutic strategy, complementing traditional treatments for WD and possibly addressing disease mechanisms linked to gut‐liver axis dysfunction.

#### 3.2.3 Effect of LBPs on Gut Microbiota at the Phylum Levels

**FIGURE 4:**
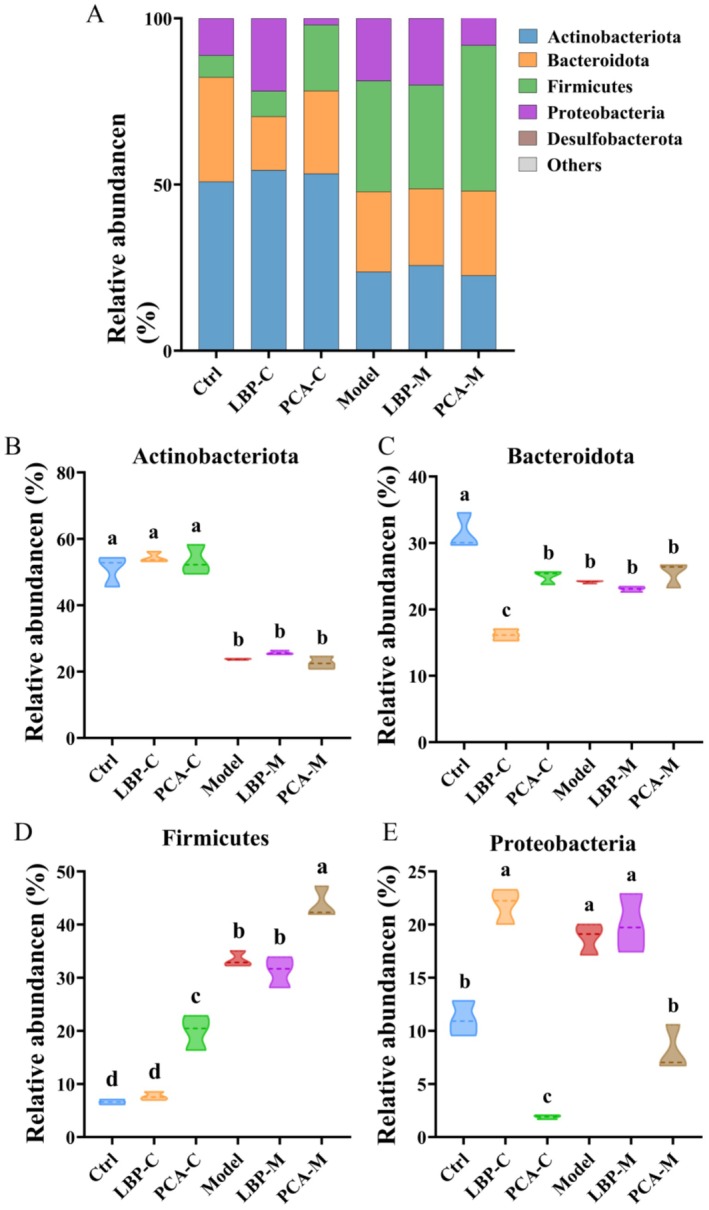
Gut microbiota composition at the phylum level across different groups. (A) Overall microbial composition. (B–E) Comparative analysis of the relative abundances of major bacterial phyla, including: (B) Actinobacteriota, (C) Bacteroidota, (D) Firmicutes, and (E) Proteobacteria. Different letters indicate significant differences among groups (*p* < 0.05), as determined by one‐way ANOVA followed by Tukey's post hoc test.

At the phylum level, gut microbiota across all groups were primarily composed of *Firmicutes*, *Actinobacteriota*, *Bacteroidota*, and *Proteobacteria* (Figure 4), which aligns with typical human gut microbial profiles (Li et al. 2024). However, notable shifts in microbial composition were observed in the Model group compared to the Control group. Specifically, the relative abundances of Firmicutes and Proteobacteria were significantly increased, while *Bacteroidota* and Actinobacteriota were markedly reduced, consistent with a dysbiotic state often associated with disease conditions such as WD. Following LBPs intervention, the LBP‐M group did not exhibit statistically significant changes in the relative abundances of these major phyla compared to the untreated Model group. This suggests that the regulatory effects of LBPs may not manifest prominently at the phylum level. Instead, their modulatory activity could be more subtle or targeted, potentially occurring at lower taxonomic levels (e.g., genus or species) or influencing functional pathways rather than broad compositional shifts. Interestingly, treatment with penicillamine resulted in a significant reduction in the relative abundance of *Proteobacteria*—a phylum often associated with inflammation and gut dysbiosis—and a concomitant increase in *Firmicutes*, suggesting a partial restoration toward a healthier microbial profile at the phylum level.

#### 3.2.4 Effect of LBPs on Gut Microbiota at the Family Levels

**FIGURE 5:**
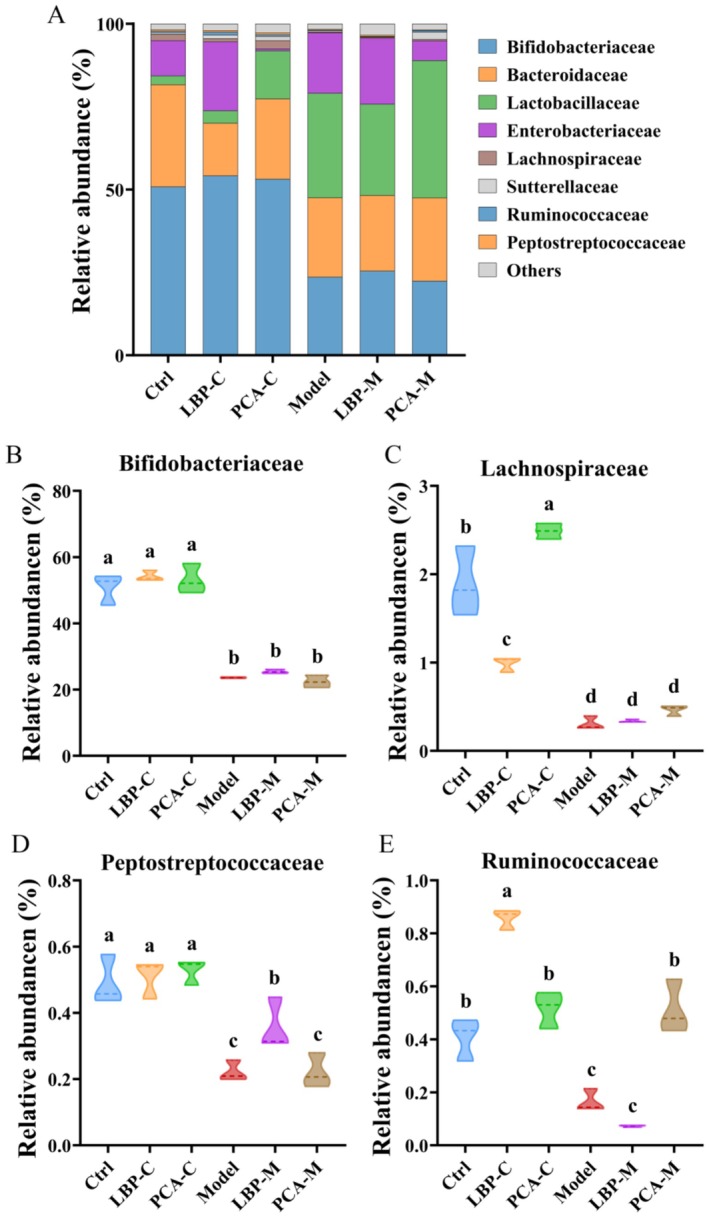
Taxonomic profiling of gut microbiota at the family level. (A) Overall bacterial composition across groups. (B–E) Comparative analysis of the relative abundances of key bacterial families, including: (B) *Bifidobacteriaceae*, (C) *Lachnospiraceae*, (D) *Peptostreptococcaceae*, and (E) *Ruminococcaceae*. Different letters indicate significant differences among groups (*p* < 0.05), as determined by one‐way ANOVA followed by Tukey's post hoc test.

At the family level, the predominant taxa identified across all groups included Bifidobacteriaceae, Bacteroidaceae, Lactobacillaceae, Enterobacteriaceae, Lachnospiraceae, Sutterellaceae, Ruminococcaceae, and Peptostreptococcaceae (Figure 5). These families represent key functional groups involved in carbohydrate fermentation, SCFAs production, and gut homeostasis. In the Model group, neither LBPs nor penicillamine treatment resulted in significant changes in the relative abundances of Bifidobacteriaceae or Lachnospiraceae, suggesting that these families may be relatively resistant to modulation under WD‐associated dysbiosis, at least within the time frame of this in vitro fermentation model. However, LBPs intervention significantly increased the abundance of Peptostreptococcaceae in the Model group (*p* < 0.05). Members of this family are known to include both commensal and potentially beneficial anaerobes capable of fermenting complex carbohydrates (Huertas‐Diaz et al. 2023), which may reflect the selective enrichment of LBP‐utilizing bacteria. In contrast, penicillamine treatment led to a significant increase in Ruminococcaceae.

In the Control group, interesting divergent effects were observed. LBPs treatment significantly decreased *Lachnospiraceae* abundance, whereas penicillamine significantly increased it. *Lachnospiraceae* is a diverse family with both beneficial and potentially pro‐inflammatory members, so the implications of these changes may depend on the specific genera affected. Additionally, LBPs significantly increased *Ruminococcaceae* in the Control group, further supporting its role in stimulating fiber‐degrading and SCFAs‐producing taxa under eubiotic conditions. These findings suggest that LBPs and penicillamine exert distinct modulatory effects on key microbial families and that their impact may vary depending on the baseline microbiota composition.

#### 3.2.5 Effect of LBPs on Gut Microbiota at the Genus Levels

**FIGURE 6:**
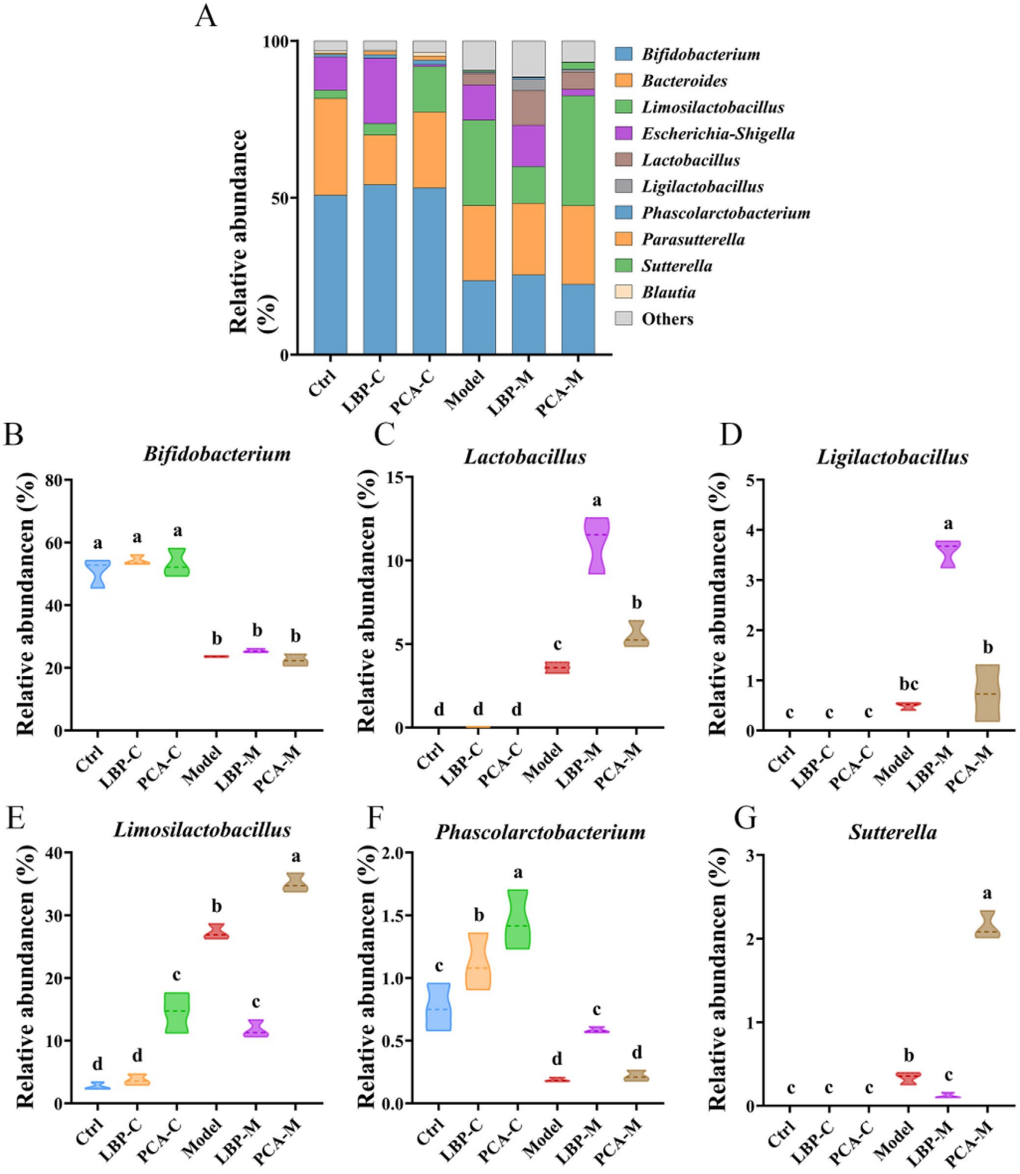
Genus level taxonomic profiling of gut microbiota. (A) Overall bacterial composition at the family level. (B–G) Comparative analysis of the relative abundances of selected genera, including: (B) *Bifidobacterium*, (C) *Lactobacillus*, (D) *Ligilactobacillus*, (E) *Limosilactobacillus*, (F) *Phascolarctobacterium*, and (G) *Sutterella*. Different letters indicate significant differences among groups (*p* < 0.05), as determined by one‐way ANOVA followed by Tukey's post hoc test.

At the genus level, the gut microbiota in all groups was predominantly composed of *Bifidobacterium*, *Lactobacillus, Bacteroides*, *Ligilactobacillus*, *Limosilactobacillus*, *Phascolarctobacterium*, and *Sutterella*, among others (Figure 6). These genera are commonly associated with host metabolic functions, immune modulation, and maintenance of gut homeostasis. As shown in Figure 6, the relative abundance of *Bifidobacterium* was significantly reduced in the Model group compared to the Control group (*p* < 0.05), reflecting a common feature of gut dysbiosis associated with WD. However, neither LBPs nor penicillamine treatment significantly restored *Bifidobacterium* levels, suggesting that this beneficial genus may be particularly sensitive to the disease‐induced microbial environment or require longer‐term interventions for recovery.

Interestingly, LBPs supplementation led to a significant increase in the relative abundances of several genera with known probiotic and anti‐inflammatory potential, including *Lactobacillus* and *Lacticaseibacillus*. These lactic acid bacteria are well‐documented for their roles in enhancing mucosal barrier integrity, modulating immune responses, and producing bioactive metabolites such as SCFAs. In the Model group, the genus *Limosilactobacillus* was abnormally elevated, a pattern sometimes associated with metabolic stress or microbial imbalance. Similarly, *Sutterella*, a genus often linked to pro‐inflammatory responses and gut barrier dysfunction, was significantly increased in the Model group. Notably, LBPs treatment significantly reduced the abundance of both *Limosilactobacillus* and *Sutterella* (*p* < 0.05), suggesting a potential role for LBPs in suppressing the overgrowth of opportunistic or contextually harmful taxa under dysbiotic conditions. In contrast, penicillamine treatment not only failed to reduce these genera but instead resulted in a significant increase in their relative abundances, further indicating that penicillamine may lack targeted microbiota‐modulating properties or could even exacerbate microbial imbalances in some cases.

Another important observation was the significant reduction of *Phascolarctobacterium* in the Model group compared to the Control. This genus is associated with SCFAs production, particularly propionate, and is considered a biomarker of gut health (Gao et al. 2025). While LBPs treatment significantly restored *Phascolarctobacterium* levels, penicillamine had no notable effect. This highlights LBP's potential to promote beneficial fermentative bacteria involved in energy metabolism and anti‐inflammatory pathways. Taken together, these results reveal that LBPs modulate the gut microbiota at the genus level in a more targeted and potentially beneficial manner than penicillamine, particularly by enriching health‐associated taxa and suppressing potentially pro‐inflammatory or dysbiosis‐associated genera. These findings underscore the therapeutic potential of LBPs as a microbiota‐directed intervention that not only improves microbial community composition but may also enhance intestinal health and host metabolic functions in the context of WD.

#### 3.2.6 Enrichment of Gut Microbiota by Lbps Identified Through LEfSe Analysis

**FIGURE 7:**
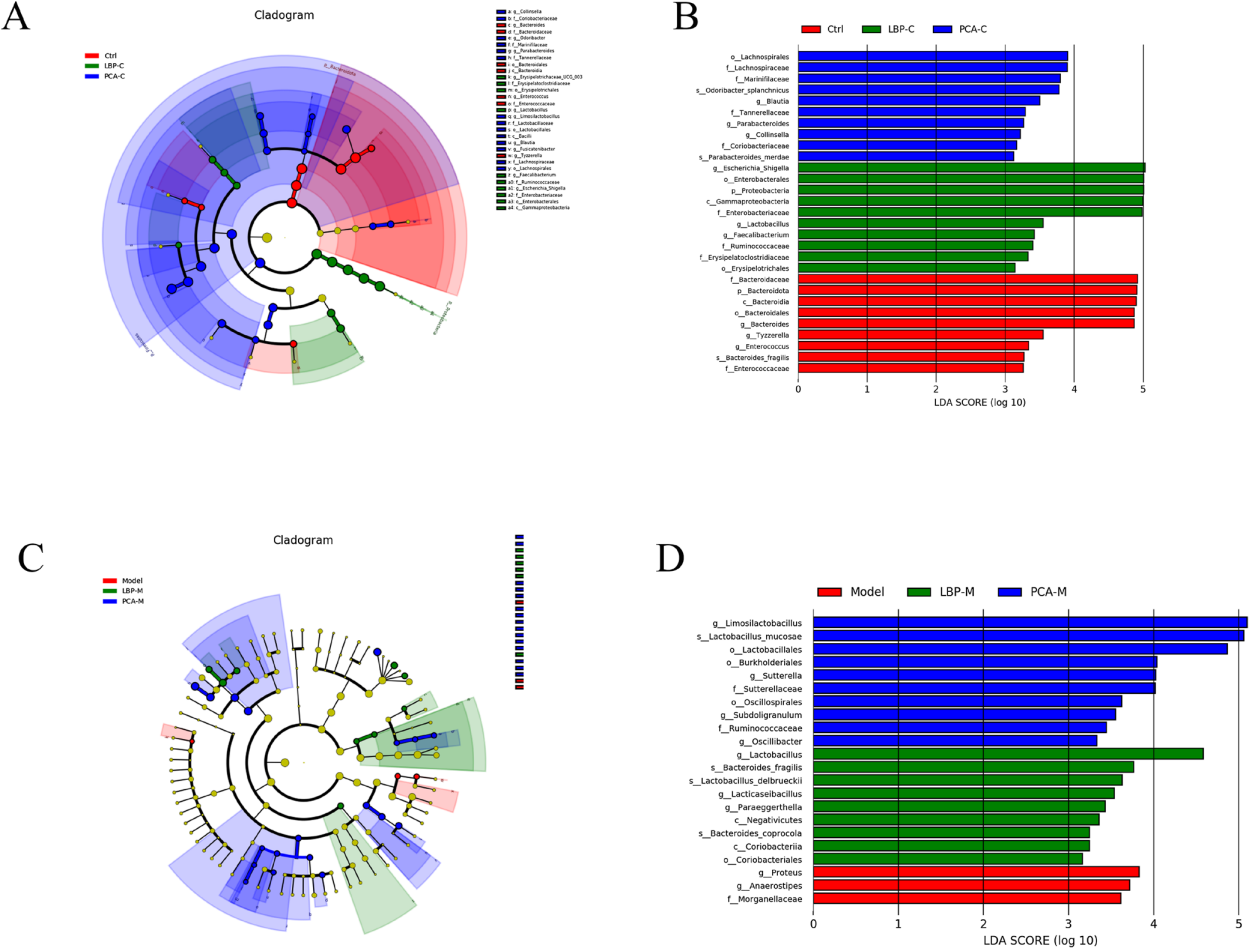
LEfSe‐based comparison of gut microbiota composition among different groups. (A, B) Differentially abundant taxa among the Ctrl, LBP‐C, and PCA‐C groups. (C, D) Differentially abundant taxa among the Model, LBP‐M, and PCA‐M groups.

To further elucidate the specific microbial taxa modulated by LBPs, Linear Discriminant Analysis Effect Size (LEfSe) analysis was performed to identify significantly enriched genera and species across treatment groups (Figure 7). In the Model group, several genera were identified as dominant biomarkers, including *Enterococcus*, *Ruminococcus*, and *Morganella*. However, following LBPs intervention, the relative abundances of these dysbiosis‐associated genera were not significantly reduced, suggesting that LBPs may not directly suppress established pathogenic taxa at the genus level. Despite this, LBPs treatment induced a distinct and beneficial enrichment of several probiotic and health‐associated taxa. Compared with the Model group, LBPs significantly increased the relative abundance of *Lactobacillus*, *Lacticaseibacillus*, and *Paraeggerthella* at the genus level. These genera are well‐documented for their roles in producing lactic acid, modulating host immunity, and maintaining intestinal barrier integrity. The expansion of *Lacticaseibacillus*, a genus recently reclassified from the traditional *Lactobacillus* clade, is particularly noteworthy, as it includes strains with advanced probiotic functions such as anti‐inflammatory activity and competitive exclusion of pathogens. At the species‐level, LBPs treatment also significantly enriched several beneficial species, including *Bacteroides fragilis* , *Bacteroides coprocola* , and *Lactobacillus delbrueckii* . Furthermore, even in the Ctrl group, LBPs supplementation led to a notable increase in *Lactobacillus*, indicating its prebiotic potential extends beyond correcting dysbiosis and may also support the maintenance of a health‐promoting microbiota in eubiotic individuals.

### 3.3 Effect of LBPs on the Production of SCFAs

As summarized in Table 2, the concentrations of major SCFAs, including acetic acid, propionic acid, n‐butyric acid, and total SCFAs, increased progressively during in vitro anaerobic fermentation across all experimental groups. This trend indicates active microbial metabolism, particularly the fermentation of carbohydrates present in the culture media. In contrast, branched‐chain fatty acids (BCFAs) such as isobutyric acid, isovaleric acid, and n‐valeric acid showed only slight increases and remained at relatively low concentrations throughout the fermentation period, suggesting that protein fermentation was minimal under the current substrate conditions. This supports the notion that the fermentation model was carbohydrate‐dominant, as intended. Before fermentation, the Model group (fecal microbiota from WD patients) exhibited significantly lower baseline concentrations of acetic acid, propionic acid, and total SCFAs compared to the Control group (healthy individuals). After fermentation, the level of SCFAs in the Control group was also much higher than that in the Model group. For instance, acetic acid levels in the Model group were 33.07 ± 1.96 mmol/L, markedly lower than those in the Control group (44.37 ± 6.16 mmol/L). This reduction reflects a compromised fermentative capacity and diminished microbial metabolic output in the context of gut dysbiosis associated with Wilson's disease, which may impair intestinal barrier function and host energy metabolism.

**TABLE 2:** Contents of SCFAs and lactic acid during in vitro fermentation.

Group	Time (h)	SCFAs (mmol/L)							
		Propionic acid	Acetic acid	i‐Butyric acid	i‐Valeric acid	n‐Butyric acid	n‐Valeric acid	Lactic acid	Total acids
Model	24	2.74 ± 0.06^b^	33.07 ± 1.96^ef^	0.06 ± 0.006^h^	0.05 ± 0.01^i^	0.57 ± 0.01^j^	0.07 ± 0.003^k^	0.48 ± 0.04^n^	37.04 ± 1.63^q^
Ctrl	24	4.11 ± 0.04^a^	44.37 ± 6.16^de^	0.06 ± 0.005^h^	0.03 ± 0.002^i^	0.61 ± 0.01^j^	0.07 ± 0.002^k^	0.86 ± 0.13^lmn^	50.12 ± 7.48^op^
LBP‐M	24	2.80 ± 0.19^b^	50.75 ± 4.11^cd^	0.06 ± 0.02^h^	0.04 ± 0.01^i^	0.63 ± 0.02^j^	0.07 ± 0.001^k^	0.53 ± 0.33^n^	54.87 ± 5.19^o^
LBP‐C	24	2.83 ± 0.12^b^	55.30 ± 1.50^c^	0.06 ± 0.01^h^	0.03 ± 0.01^i^	0.64 ± 0.04^j^	0.06 ± 0.001^k^	0.52 ± 0.14^n^	59.45 ± 1.97^o^
PCA‐M	24	2.48 ± 0.08^b^	34.12 ± 0.93^f^	0.06 ± 0.01^h^	0.05 ± 0.01^i^	0.58 ± 0.03^j^	0.07 ± 0.002^k^	0.73 ± 0.18^mn^	38.11 ± 1.07^q^
PCA‐C	24	2.90 ± 0.02^b^	41.43 ± 1.38^ef^	0.06 ± 0.01^h^	0.04 ± 0.01^i^	0.56 ± 0.01^j^	0.09 ± 0.02^k^	0.52 ± 0.35^n^	45.57 ± 1.01^pq^

*Note:* Values are expressed as mean ± SD (*n* = 3). Different superscript letters within the same column indicate significant differences among time points (*p* < 0.05), determined by one‐way ANOVA followed by Tukey's post hoc test.

Abbreviation: BF, before fermentation.

After 24 h of fermentation, distinct changes were observed in response to LBPs and penicillamine treatments. In the LBP‐M group, the production of acetic acid, n‐butyric acid, and total SCFAs increased significantly compared to the untreated Model group (*p* < 0.05). Specifically, acetic acid levels increased from 33.07 ± 1.96 mmol/L to 50.75 ± 4.11 mmol/L, representing a ~53% elevation in acetic acid output. Notably, this level even surpassed that of the healthy Control group (44.37 ± 6.16 mmol/L), indicating that LBPs supplementation enhanced the metabolic performance of the dysbiotic microbiota beyond baseline eubiotic levels.

In contrast, penicillamine treatment (PCA‐M) did not significantly improve SCFAs production, with acetic acid reaching only 34.12 ± 0.93 mmol/L, a level comparable to the untreated Model group. This suggests that, while penicillamine may offer therapeutic benefits through copper chelation, it lacks the capacity to restore microbial metabolic activity or promote beneficial fermentation processes. In the healthy Control group, similar patterns were observed. LBPs supplementation (LBP‐C) further elevated acetic acid levels to 55.30 ± 1.50 mmol/L, exceeding both the Control and penicillamine‐treated groups (PCA‐C: 41.43 ± 1.38 mmol/L). This reinforces the notion that LBPs act as an efficient prebiotic substrate, stimulating the proliferation and activity of SCFA‐producing taxa, even in an already balanced microbial environment.

## 4 Discussion

GDFMD has been widely used in the clinical treatment of WD and has demonstrated promising efficacy in both patients and animal models (Wei et al. 2021). *L. barbarum* , the principal component (“monarch drug”) of GDFMD, is commonly used in both food and pharmaceutical applications. Among its bioactive compounds, LBPs are considered the major functional ingredients, noted for their physicochemical stability and fermentability during gastrointestinal digestion. Previous studies have shown that LBPs exhibit strong resistance to enzymatic degradation in simulated digestion, with minimal changes in molecular weight, monosaccharide composition, and reducing sugar content (Wang et al. 2024). This digestive resistance allows LBPs to reach the colon largely intact, where they can be metabolized by gut microbiota, thereby functioning as prebiotics. Their anti‐digestive properties enable LBPs to maintain bioactivity and selectively stimulate beneficial bacteria, suggesting their potential to modulate gut microbial structure and improve host health.

WD is a genetic disorder resulting from mutations in the ATP7B gene, leading to copper accumulation primarily in the liver and brain. Excessive copper accumulation in WD may alter the intestinal microenvironment through oxidative stress, epithelial injury, and immune dysregulation, thereby disturbing gut microbial composition (Ala et al. 2007; Sturniolo et al. 1999). Conversely, microbiota‐derived metabolites may modulate intestinal barrier function and copper homeostasis, suggesting a bidirectional interaction between WD and gut dysbiosis (Tilg et al. 2020). Recent studies have revealed significant gut microbiota dysbiosis in WD patients, characterized by reduced diversity, depletion of beneficial bacteria, and enrichment of pathogenic taxa (Cai et al. 2024; Zhong et al. 2024). In our study, PCoA and clustering revealed distinct microbial profiles in the WD (Model) group compared to healthy controls, supporting the existence of a WD‐specific gut microbial signature. Our findings demonstrated that LBPs supplementation significantly altered the gut microbiota composition of WD patients. The result revealed that LBPs selectively modulate the gut microbiota at the genus level by enriching health‐associated taxa and suppressing dysbiosis‐related genera, demonstrating a more targeted and beneficial effect than penicillamine. LBPs supplementation significantly increased the relative abundances of several probiotic genera, notably *Lactobacillus*, *Lacticaseibacillus*, and *Paraeggerthella*. These bacteria are well known for their lactic acid and SCFAs production, immune‐modulatory properties, and barrier‐supportive functions (Lemos Junior et al. 2025). For instance, *Lacticaseibacillus*, a recently reclassified genus from the *Lactobacillus* clade, includes strains with strong anti‐inflammatory activity and the ability to competitively inhibit pathogenic colonization (Cui and Qu 2021). The intestinal flora may influence the metabolism of copper ions by regulating their absorption and excretion (Hasr Moradi Kargar and Hadizadeh Shirazi 2020). *Lactobacillus* binds copper ions and reduces their absorption, thereby reducing their accumulation in the body (Tian et al. 2015). Hepatomegaly (Wilson disease) is an autosomal recessive disorder caused by mutations in the ATP7B gene, resulting in impaired copper ion metabolism and abnormal deposition of copper ions in the liver, brain and other tissues. In recent years, it has been found that intestinal microorganisms may play an important role in the metabolism of copper ions and the pathologic process of hepatomegaly. Copper ion deposition and intestinal flora dysbiosis may lead to impaired intestinal barrier function, increased endotoxin and pathogenic bacterial translocation, and further exacerbation of hepatic and systemic inflammatory responses (Zhao et al. 2024). Their expansion in the LBP‐M group highlights the potential of LBPs to promote a probiotic‐dominant intestinal environment.

Additionally, *Phascolarctobacterium*, a genus associated with propionate production and maintenance of metabolic homeostasis (Gao et al. 2025; Liebana‐Garcia et al. 2025), was significantly reduced in the Model group but restored following LBPs intervention. This suggests that LBPs not only promote the growth of traditional probiotics but also stimulate functionally relevant fermentative bacteria involved in energy metabolism and anti‐inflammatory pathways. In contrast, penicillamine failed to restore this genus, further emphasizing the metabolically targeted effects of LBPs. Interestingly, while the abundance of *Bifidobacterium*, a hallmark genus of gut health, was significantly reduced in WD microbiota, neither LBPs nor penicillamine restored its levels. The lack of restoration of Bifidobacterium may be attributed to several factors. First, Bifidobacterium is highly sensitive to intestinal environmental conditions, and its recovery may require longer intervention periods or specific prebiotic substrates that selectively promote its growth. Second, both LBPs and penicillamine primarily exert their effects through metal chelation and modulation of oxidative stress and inflammation rather than directly stimulating the proliferation of *Bifidobacterium*. In addition, competitive interactions with other gut microbial taxa that were preferentially enriched by these treatments may have limited the recolonization of *Bifidobacterium*.

In parallel with the enrichment of beneficial taxa, LBPs also reduced the abundance of contextually harmful or opportunistic genera, such as *Limosilactobacillus* and *Sutterella*, both of which were elevated in the Model group. *Limosilactobacillus* overgrowth has been associated with metabolic stress (Aguilar et al. 2020; Chen et al. 2021), while *Sutterella* has been linked to gut barrier dysfunction and pro‐inflammatory responses (Dupraz et al. 2025; Wang et al. 2013). LBPs significantly suppressed both genera, whereas penicillamine not only failed to reduce them but resulted in a notable increase, suggesting that LBPs may offer protective effects against dysbiosis‐associated inflammation, which traditional copper‐chelating therapies lack.

Moreover, LEfSe analysis revealed that LBPs significantly enriched several species‐level probiotics, including *B. fragilis* , *B. coprocola* , and *L. delbrueckii* . *B. fragilis* , in particular, is known for its role in regulating T‐cell–mediated immune responses and producing beneficial SCFAs, further supporting the immunomodulatory potential of LBPs (Chen et al. 2024; Zhou et al. 2022). The ability of LBPs to increase these species even in healthy (Control) microbiota indicates that its prebiotic effects are not limited to disease correction, but can also enhance microbial function in eubiotic conditions. *L. delbrueckii* is a well‐characterized probiotic species widely used in fermented dairy products, such as yogurt and kefir (Olorocisimo et al. 2023; Sun et al. 2023). It is known for its robust lactic acid production, ability to lower gut pH, and capacity to enhance mucosal barrier function. Moreover, *L. delbrueckii* has demonstrated anti‐inflammatory properties and the ability to modulate immune responses, making it a valuable member of health‐promoting gut microbiota (de Jesus et al. 2024). Its enrichment in the LBP‐treated group suggests that LBPs may help restore a probiotic‐rich environment that supports gut integrity and host immune homeostasis. *B. coprocola* , while not traditionally classified as a probiotic, is a commensal species commonly detected in healthy individuals. Recent studies have suggested that members of the *Bacteroides* genus, including *B. coprocola* , engage in the fermentation of dietary fibers and contribute to the production of beneficial metabolites such as SCFAs (Qu et al. 2025). Although its role is less well‐defined compared to *B. fragilis* , the enrichment of *B. coprocola* may reflect an enhanced capacity for carbohydrate metabolism and overall microbial fermentative activity following LBPs supplementation. Further studies are warranted to clarify its functional contributions in the context of gut health and disease.

SCFAs are the main metabolites of dietary fiber fermentation by the gut microbiota, and they play a key role in maintaining intestinal health and overall metabolic homeostasis, including acetic acid, propionic acid, n‐butyric acid, isobutyric acid, n‐valeric acid, isovaleric acid, and lactic acid, which are the end‐products of dietary fiber produced by anaerobic gut microbial fermentation (Facchin et al. 2024). SCFAs and other metabolites produced by the intestinal microbiota may indirectly influence the metabolism and deposition of copper ions by regulating intestinal barrier function and immune responses. These metabolites can provide energy to maintain intestinal barrier integrity, prevent harmful substances from entering the systemic circulation, and modulate host immune responses, thereby contributing to immune homeostasis (Dalile et al. 2019). Influence gene expression patterns and participate in the regulation of inflammation, lipid metabolism and other physiological processes Thus, SCFAs play a key role in human health (Campos‐Perez and Martinez‐Lopez 2021). The results showed that model group had significantly lower levels of acetic acid, and total acid before fermentation compared with Control group, and this result may be related to the pathological state of Model group. SCFAs (especially acetic acid, propionic acid and butyric acid) are important metabolites produced by intestinal flora in fermentation of dietary fibers, and they have the ability to maintain the intestinal barrier function, regulating immune response and inhibiting inflammation. Compared with the Model group, the LBP‐M group showed a significant increase in the content of acetic acid, n‐butyric acid and total acid after 24 h of anaerobic fermentation in vitro, which indicated that LBP‐M could significantly improve the production of SCFAs in the Model group. LBPs may increase the production of SCFAs this with gut microorganisms by selectively promoting the growth of probiotic bacteria such as *Lactobacillus, Lacticaseibacillus*, and enhancing their fermentation ability. This is consistent with the results of the preliminary analysis of LeFSe. LBPs may increase the production of SCFAs such as acetic acid and butyric acid by modulating the metabolic pathways of the flora. The significant increase in isobutyric, lactic and total acids in the LBP‐C group compared to the Control group is a result that suggests that LBPs is able to significantly modulate the metabolic function of the intestinal flora and increase the production of specific SCFAs, even in a healthy state. This finding further supports the prebiotic properties of LBPs, suggesting that it is not only capable of improving intestinal health in pathological states, but also enhancing the metabolic functions of intestinal flora in healthy states. Compared with the Model group, the differences in the content of SCFAs in the Penicillamine Model group were not statistically significant except for propionic acid. This result suggests that Penicillamine, a drug commonly used in the treatment of hepatoblastoid nuclear degeneration, has a limited modulating effect on the production of SCFAs, which may be related to its lesser effect on the intestinal flora. This finding suggests that *Penicillamine* may exert its therapeutic effects mainly through other mechanisms (e.g., copper chelation) and has a weak modulatory effect on the metabolic functions of the intestinal flora.

## 5 Conclusion

In summary, this study demonstrates that LBPs possess significant prebiotic potential in the context of WD. Using an in vitro fermentation model, we revealed that the gut microbiota of WD patients exhibits reduced microbial diversity and impaired SCFAs production compared to healthy individuals. Notably, LBPs supplementation effectively modulated the gut microbial composition by enriching beneficial and SCFA‐producing taxa and significantly enhanced the levels of acetic acid and total SCFAs, outperforming the conventional chelating agent penicillamine. These findings suggest that LBPs may restore microbial metabolic function in WD through microbiota‐mediated pathways and highlight their potential as a novel microbiota‐targeted adjunctive therapy. Further in vivo studies and clinical investigations are warranted to validate these effects and to explore the underlying mechanisms by which LBPs exert their therapeutic benefits in WD.

## Author Contributions

**Xiang Fang:** validation. **Yulong Yang:** visualization. **Kangyi Zhang:** visualization, software, project administration. **Shuzhen Fang:** conceptualization, writing – original draft, methodology, data curation. **Danqing Liu:** investigation. **Wenming Yang:** writing – review and editing, resources, funding acquisition, supervision. **Guijie Chen:** writing – review and editing, writing – original draft.

## Funding

This work was supported by the Major Science and Technology Project of the National Natural Science Foundation Regional Innovation Development Joint Fund Project, U22A20366. Anhui Province Traditional Chinese Medicine Technology Resea, 202303a07020004. Anhui Higher Education Collaborative Innovation Project, GXXT‐2020‐025. Anhui Province Clinical Medicine Research and Transformation Special Project, 202204295107020066.

## Conflicts of Interest

The authors declare no conflicts of interest.

## Data Availability

The data that support the findings of this study are available on request from the corresponding author. The data are not publicly available due to privacy or ethical restrictions.
